# The protein degradation system encoded by *hslUV* (*ClpYQ*) is dispensable for the virulence of *Haemophilus ducreyi* in human volunteers

**DOI:** 10.1128/iai.00577-24

**Published:** 2025-04-10

**Authors:** Kate R. Fortney, Julie A. Brothwell, Teresa A. Batteiger, Rory Duplantier, Barry P. Katz, Stanley M. Spinola

**Affiliations:** 1 Department of Microbiology and Immunology, Indiana University School of Medicine, Indiana University 1772, Indianapolis, Indiana, USA; 2 Department of Medicine, Indiana University School of Medicine, Indiana University 196273 , Indianapolis, Indiana, USA; 3 Department of Biostatistics and Health Data Science, Indiana University School of Medicine, Indiana University 1772, Indianapolis, Indiana, USA; 4 Department of Pathology and Laboratory Medicine, Indiana University School of Medicine, Indiana University 464871 , Indianapolis, Indiana, USA; University of Illinois Chicago, Chicago, Illinois, USA

**Keywords:** *Haemophilus ducreyi*, *hslUV (clpYQ)*, human infection, proteases

## Abstract

*Haemophilus ducreyi* causes cutaneous ulcers in children who live in yaws-endemic countries and the genital ulcer disease chancroid. In the human host, *H. ducreyi* resides in an abscess and may need to resist both heat and oxidative stress, which result in aggregation and misfolding of bacterial proteins. In *Escherichia coli*, the *hslUV* (*clpYQ*) operon encodes a proteasome-like complex that degrades misfolded proteins and is upregulated during heat shock. In previous studies, we showed that *hslUV* transcripts are upregulated in experimental lesions caused by *H. ducreyi* in human volunteers, suggesting that HslUV may help *H. ducreyi* adapt to the abscess environment. Here, we constructed an unmarked *hslUV* operon deletion mutant, 35000HPΔ*hslUV*, in *H. ducreyi*. Whole-genome sequencing showed that compared to its parent (35000HP), the mutant contained only the deletion of interest. Six volunteers were inoculated at three sites on skin overlying the deltoid on opposite arms with 35000HP and 35000HPΔ*hslUV*. Within 24 h, papules formed at 88.9% (95% CI [69%, 100%]) at both parent and mutant-inoculated sites (*P* = 1.0). Pustules formed at 44.4% (95% CI [25.6%, 64.3%]) at parent-inoculated sites and 33.3% (95% CI [2.5%, 64.1%]) at mutant-inoculated sites (*P* = 0.17). Thus, the proteosome-like complex encoded by *hslUV* was dispensable for *H. ducreyi* virulence in humans. In the absence of *hslUV*, *H. ducreyi* likely utilizes other systems such as the Lon protease, ClpXP, and ClpB/DnaK to combat protein aggregation and misfolding, underscoring the importance of the functional redundancy of such systems in gram-negative pathogens.

## INTRODUCTION

*Haemophilus ducreyi* causes chancroid, a genital ulcer disease that facilitates the transmission of the human immunodeficiency virus (HIV)-1 (1). *H. ducreyi* is thought to enter the host through breaks in the epithelium that occur during intercourse. Erythematous papules form at entry sites within hours to days and evolve into pustules in 2–3 days (2). After ~2 weeks, the pustules ulcerate, and patients typically have one to four ulcers. The clinical reservoir of chancroid is infected sex workers. Through targeted antimicrobial prophylaxis of sex workers and syndromic management of genital ulcers, the global prevalence of chancroid has dramatically declined, but chancroid remains endemic in certain African countries (1, 3, 4). Although long thought to be exclusively sexually transmitted, *H. ducreyi* was identified as a major cause of painful skin ulcers in children who live in yaws-endemic countries of the South Pacific, Asia, and Africa (1, 5). The latter infections are likely due to traumatic breaks in the skin that occur in colonized individuals or subsequent contact of wounds with environmental *H. ducreyi* reservoirs (1, 6–8). Because *H. ducreyi* can asymptomatically colonize the skin and reside in the environment, cutaneous ulcers due to *H. ducreyi* are not likely to be eradicated by antimicrobial therapy (1). Thus, *H. ducreyi* infections remain an important problem for global health.

To study *H. ducreyi* pathogenesis, we developed an infection model of human volunteers (reviewed in references 1, 9–11). In this model, healthy adults are inoculated with *H. ducreyi* strain 35000HP (HP, human passaged) delivered via puncture wounds at multiple sites on the skin overlying the deltoid. Within 24 h of inoculation with an estimated delivered dose (EDD) of ~1–150 CFU, papules form at inoculated sites, and either spontaneously resolve or evolve into pustules 2–5 days later (10). Due to safety considerations, the duration of experimental infection is limited to the pustular stage of disease. In both experimental pustules and natural chancroid, *H. ducreyi* is found in an abscess and co-localizes with macrophages and neutrophils, which fail to ingest the organism (12, 13). Thus, in both natural and experimental infections of humans, *H. ducreyi* must adapt to the hostile environment within the abscess to survive.

By residing in an abscess, *H. ducreyi* encounters oxidative stress in the form of reactive oxygen species *in vivo*. Heat may be another stress that the organism faces *in vivo*, since *H. ducreyi* grows optimally at 33°C and dies at 37°C *in vitro* (1, 14). Both oxidative and heat stress result in the aggregation or misfolding of bacterial proteins, which can lead to cell death. To ensure bacterial survival, aggregated or misfolded proteins are usually targeted by protein quality control systems for refolding or removal from the bacterial cell (reviewed in references 15–17). The most common degradation systems are the energy-dependent ATPases associated with various cellular activities (AAA+)-protease complexes, which usually consist of one component that binds ATP and directs the misfolded protein to the proteolytic chamber of a second component (16); however, Lon protease contains both of these activities (18). Another AAA+ member, ClpB, and DnaK form the canonical system that refolds damaged proteins that aggregate in response to heat (15, 17). Of these protein quality control systems, the 35000HP chromosome contains homologs of *hslUV* (*clpYQ*), *clpXP*, *clpB-dnaK*, and *lon* (Table S1).

We recently used RNA-seq to determine differentially expressed *H. ducreyi* transcripts in pustules compared to the inoculum (i.e., mid-log phase bacteria) used to infect the volunteers. In two independent studies, transcripts corresponding to *hslUV* were significantly upregulated (>2-fold change) in pustules compared to the inoculum (19, 20). We therefore hypothesized that *hslUV* may be essential for *H. ducreyi* virulence in humans.

Here, we characterized the *hslUV* operon of *H. ducreyi*, constructed a *hslUV* deletion mutant, and tested the mutant for virulence in human volunteers. We also compared the ability of the mutant and its isogenic parent to survive heat shock and oxidative stress *in vitro*.

## RESULTS

### *H. ducreyi hslU* and *hslV* are members of an operon

**Fig 1:**
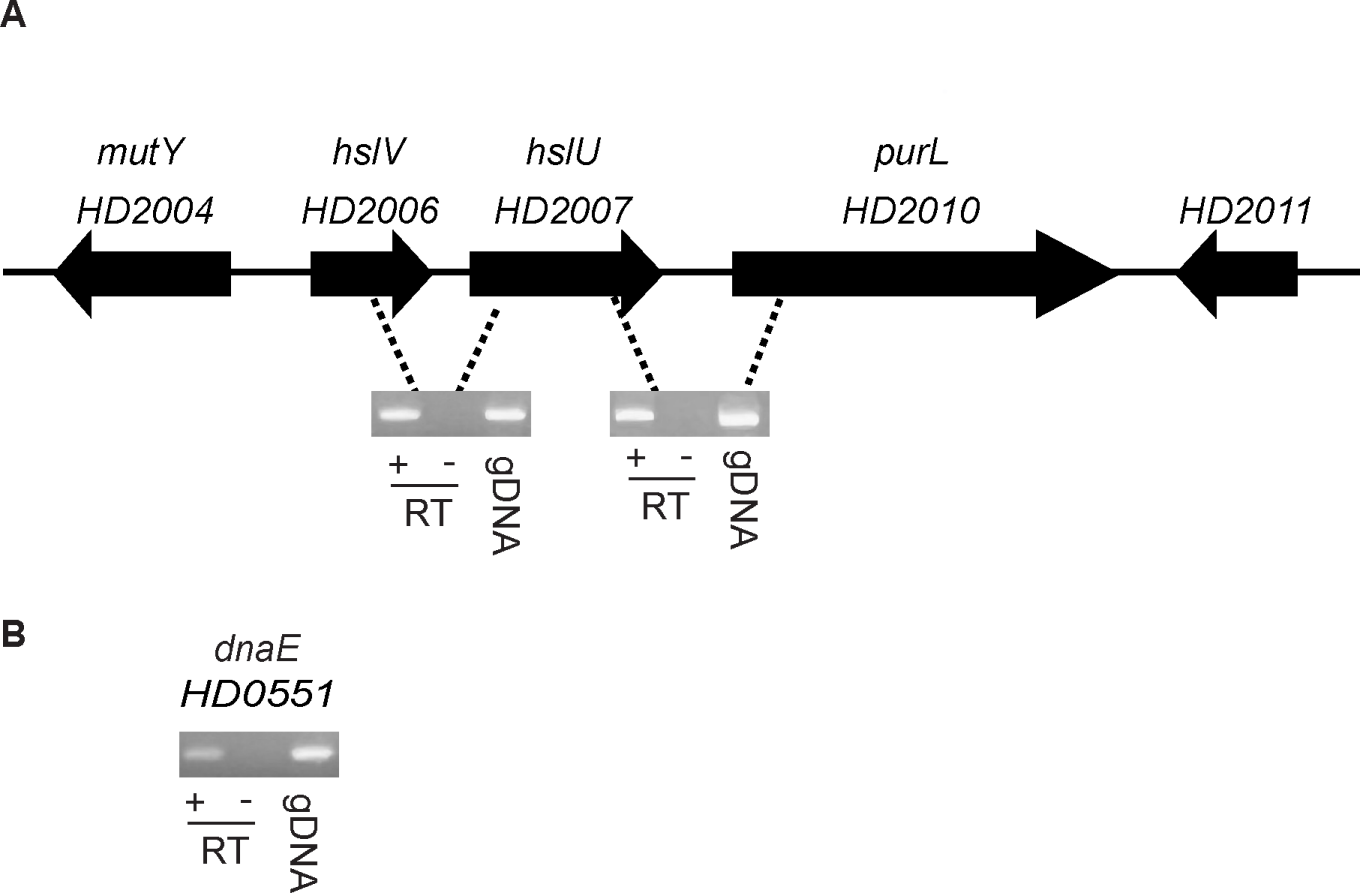
*hslUV* forms an operon with *purL*. (**A**) RT-PCR of the junctions between *hslV, hslU,* and *purL* show that the three genes are co-transcribed. (**B**) Reverse transcriptase of *dnaE* served as a positive control for RT-PCR reactions. A representative gel from three individual experiments is shown. gDNA, genomic DNA control; RT+, reactions with reverse transcriptase; RT−, without reverse transcriptase.

In *H. ducreyi* strain 35000HP, *hslUV* are in a putative operon that spans HD2006 to HD2010 (Fig. 1A). To determine if *hslU*, *hslV*, and the downstream gene, *purL*, were in an operon, we performed RT-PCR on aerobically grown mid-log phase cultures of 35000HP using primers (Table S2) that span the gene junctions. As controls, we included reactions that lacked the reverse transcriptase or contained genomic DNA (Fig. 1B). *hslU* and *hslV* were co-transcribed with *purL* (Fig. 1A).

### *hslUV* is dispensable for virulence in humans

**Fig 2:**
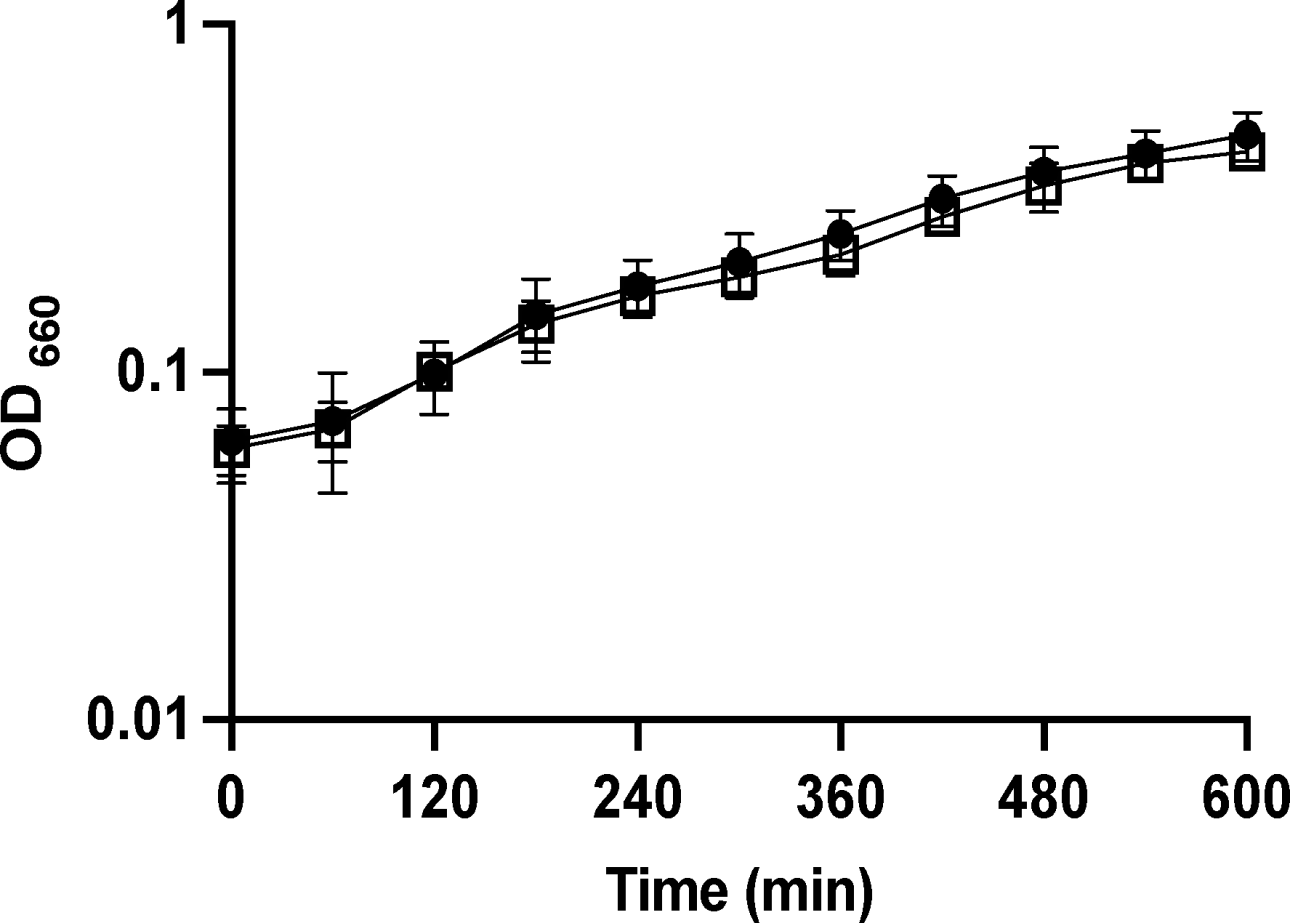
Growth of 35000HP (circles) and 35000HP∆*hslUV* (squares) in GC broth supplemented with 1% IsoVitaleX, 5% heat-inactivated fetal bovine serum, and 50 µg/mL hemin. Growth was measured by optical density (OD_660_) in three independent experiments. Error bars represent standard deviation. No significant differences were found by two-way repeated measures ANOVA.

*hslUV* transcripts are significantly upregulated in experimental lesions caused by 35000HP (19, 20). To investigate the role of *hslUV* in 35000HP virulence*,* we constructed an unmarked, in-frame deletion mutant using recombineering technology (21, 22). Whole-genome sequencing showed that the 35000HP∆*hslUV* mutant contained the expected deletion, but did not contain any other mutations. By RT-PCR, *purL* was still transcribed in the mutant (data not shown). 35000HP and 35000HP∆*hslUV* displayed similar growth kinetics in the proteose peptone-based media that is used to grow the human challenge inoculum (Fig. 2).

Per protocol, we attempted to inoculate a group of three volunteers with EDDs of 45, 90, and 180 CFU of the mutant at three sites on the upper arm and 90 CFU of the parent at three sites on the opposite arm. Three volunteers were inoculated with EDDs of 38, 76, and 151 CFU of the mutant and 72 CFU of the parent (Table 1). Papules formed at all nine mutant and all nine parent-inoculated sites; pustules formed at three out of nine mutant-inoculated sites and five out of nine parent-inoculated sites (Table 1). One pustule spontaneously resolved at endpoint in volunteer 508.

**TABLE 1:** Response to inoculation with live *H. ducreyi*

Volunteer^*a*^ (gender)	Observation period (days)	Strain^*b*^	EDD (CFU)^*c*^	No. of initial papules	No. of initial pustules	No. of pustules at endpoint
508 (F)	7	P	72	3	2	1
		M	38–151	3	1	1
512 (F)	7	P	72	3	1	1
		M	38–151	3	0	0
514 (M)	7	P	72	3	2	2
		M	38–151	3	2	2
515 (M)	7	P	113	3	2	2
		M	26–102	3	3	3
516 (M)	7	P	113	3	1	1
		M	26–102	3	0	0
517 (F)	6	P	113	1	0	0
		M	26–102	1	0	0

^*a*^ Volunteers 508, 512, and 514 were inoculated in the first iteration; 515, 516, and 517 in the second iteration. F, female; M, male.

^*b*^ P, parent strain 35000HP; M, mutant strain 35000HP∆*hslUV.*

^*c*^ EDD, estimated delivered dose; 38–151, one dose each of 38, 76, and 151 CFU; 26–102, one dose each of 26, 50, and 102 CFU.

As the mutant formed pustules at doses similar to the parent, data from the initial group suggested the mutant did not have a virulence defect. Per protocol, we repeated the experiment in a second group of three volunteers who were inoculated with EDDs of 26, 51, and 102 CFU of the mutant and 113 CFU of the parent. Papules formed at seven out of nine mutant-inoculated and seven out of nine parent-inoculated sites; pustules formed at three mutant-inoculated sites and three parent-inoculated sites (Table 1). Since the mutant formed pustules in the second group of volunteers, the trial was stopped per protocol.

Overall, papules formed at 88.9% (95% CI [69%, 100%]) at both 35000HP and 35000HP∆*hslUV-*inoculated sites (Table 1). After 24 h of infection, the mean ± SD area of papules at parent sites was 9.1 ± 8.5 and 6.2 ± 8.0 mm^2^ at mutant sites (*P* = 0.17). Pustules formed at 44.4% (95% CI [25.6%, 64.3%]) of parent-inoculated sites and 33.3% (95% CI [2.5%, 64.1%]) at mutant-inoculated sites (*P* = 0.17). Thus, *hslUV* was dispensable for pustule formation in the model.

Three volunteers (508, 514, and 515) developed pustules at both mutant and parent-inoculated sites, while two volunteers (512 and 516) developed pustules only at parent-inoculated sites (Table 1). One volunteer (517) did not develop or resolved infection at all inoculated sites (Table 1). Biopsies from one parent (*N* = 5) and one mutant (*N* = 3) site were acquired from the five volunteers who formed pustules. The biopsies were divided in half and semi-quantitatively cultured or fixed in formalin and stained with hematoxylin-eosin and anti-CD3 antibodies as described (23). The parent and mutant specimens were indistinguishable and both contained abscesses composed primarily of neutrophils that had eroded through the epidermis. There was a dense monocytic perivascular and interstitial infiltrate in the dermis; the dermal infiltrate consisted primarily of CD3^+^ cells. These findings are typical of experimental pustules (24).

Of the five biopsy specimens cultured from parent sites, four yielded *H. ducreyi*; the three mutant-inoculated sites all yielded *H. ducreyi*. The number of viable *H. ducreyi* recovered from the parent sites was 2.0 × 10^5^ ± 2.5 × 10^5^ (mean ± SD) CFU/g tissue and from mutant sites was 5.4 × 10^5^ ± 6.2 × 10^5^ CFU/g tissue (*P* = 1.0).

To examine whether cross-contamination had occurred between the parent and the mutant inocula and/or the parent- and the mutant-inoculated sites, we tested a minimum of 30 colonies isolated from each of the inocula and all colonies isolated from the daily surface cultures and biopsies for the presence of *hslUV* by colony hybridization. As a positive control for both bacterial strains, we used a probe against *dnaE*. The *dnaE* probe (Table S2) hybridized to all colonies tested from the parent (*N* = 72) and mutant (*N* = 72) inocula, while the *hslUV* probe (Table S2) only hybridized to colonies from the parent inocula. At least one positive surface culture for *H. ducreyi* was obtained during follow-up visits from 27.8% of the parent-inoculated and 22.2% of the mutant-inoculated sites. The *hslUV* probe hybridized to all colonies recovered from surface cultures of parent sites (*N* = 187) and none of the colonies recovered from surface cultures of mutant sites (*N* = 188); the *dnaE* probe hybridized to all colonies recovered from surface cultures of both parent and mutant sites (*N* = 375). The *dnaE* probe hybridized to all colonies recovered from biopsies of parent (*N* = 143) and mutant (*N* = 108) sites; the *hslUV* probe hybridized only to the colonies recovered from biopsies of parent sites. Thus, there was no evidence of cross-contamination between the mutant and parent inocula or inoculation sites.

### Deletion of *hslUV* does not affect *H. ducreyi* resistance to oxidative stress or heat shock *in vitro*

**Fig 3:**
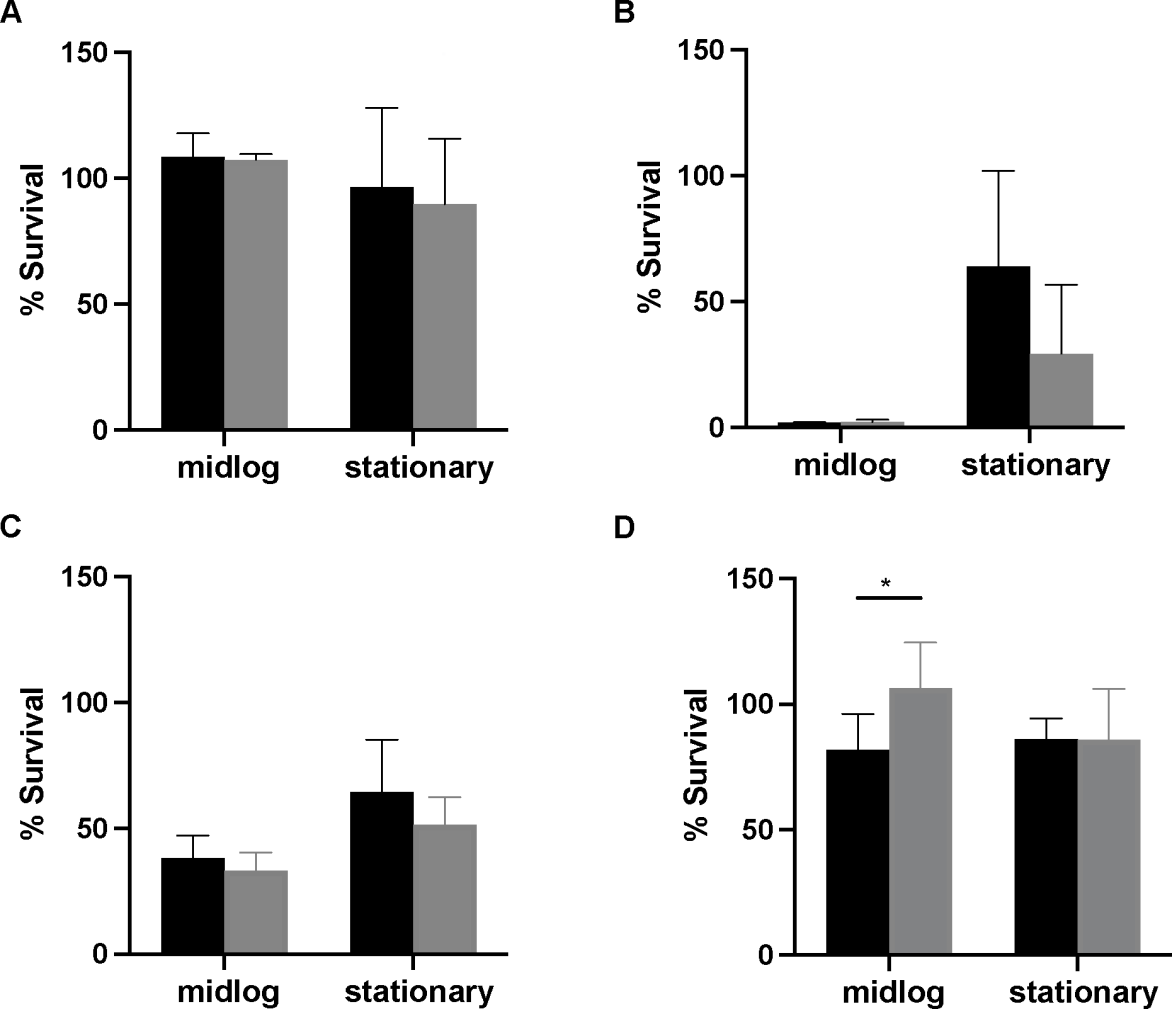
Survival of 35000HP (black bars) and 35000HP∆*hslUV* (gray bars) after treatment with (**A**) 0.2 mM paraquat, (**B**) 2.0 mM paraquat, (**C**) 2.0 mM H_2_O_2_, or (**D**) heat shock at 40°C. Survival was measured by colony-forming units (CFU) pre- and post-treatment in four independent experiments. Percent survival was calculated by determining the ratio of CFU recovered after treatment to CFU recovered prior to treatment. Error bars represent standard deviation. Statistically significant differences were determined by mixed model analysis of variance (ANOVA) followed by Tukey’s honestly significant difference test. *, *P* < 0.05.

Since *hslUV* was upregulated and 35000HP∆*hslUV* could survive as well as 35000HP in human infection, we next determined whether *hslUV* had a role in bacterial survival in response to oxidative stress. We compared the survival of 35000HP and 35000HP∆*hslUV* grown to mid-log and stationary phase after exposure to paraquat, which enhances superoxide production, or H_2_O_2_, as described previously (25). When bacteria were grown to mid-log or stationary phase and treated with 0.2 mM paraquat, 2.0 mM paraquat, or 2.0 mM H_2_O_2_ for 1 h, there were no significant differences in the survival of the parent and mutant strains (Fig. 3).

In *E. coli* and other organisms, HslUV removes misfolded proteins induced by heat shock (16, 26). To determine if *hslUV* has a role in the heat shock response of *H. ducreyi*, we grew the mutant and parent to mid-log and stationary phase at 33°C, which is the optimal temperature for *H. ducreyi* growth. We compared the survival of 35000HP and 35000HP∆*hslUV* after 2 h of incubation at 40°C. The survival of the mutant (106.6 ± 18%) was significantly higher than the parent (81.8 ± 14.4%) for the mid-log phase organisms (*P* = 0.042), but there was no difference in the survival of the strains in stationary phase (*P* = 0.99) (Fig. 3). Thus, *hslUV* did not play a major role in the survival of *H. ducreyi* during oxidative stress or heat shock *in vitro*.

## DISCUSSION

We had previously shown that *H. ducreyi* mutants that lack the expression of carbon storage regulator A (CsrA), guanosine pentaphosphate and tetraphosphate ((p)ppGpp), or the DnaK suppressor protein (DksA) have significantly reduced ability to tolerate oxidative stress and/or heat shock and are partially attenuated for pustule formation in human volunteers (25, 27, 28). Since resistance to oxidative stress and heat shock may play a critical role in *H. ducreyi* pathogenesis and since transcripts corresponding to the *hslUV* protein quality control system are upregulated in experimental lesions (19, 20), we compared the virulence of 35000HPΔ*hslUV* to that of 35000HP in humans. We found that 35000HPΔ*hslUV* formed pustules at a similar rate to the parent and was virulent. In addition, the mutant was not more susceptible to oxidative stress or heat shock than its isogenic parent *in vitro*. Thus, in the absence of *hslUV*, *H. ducreyi* likely uses other mechanisms such as CsrA, (p)ppGpp, DksA, ClpXP, ClpB-DnaK, and Lon to combat these stresses. Of these systems, *csrA* and *dnaK* transcripts are also upregulated in pustules (19) and perhaps compensate for the deletion of *hslUV*.

Although protein degradation systems such as ClpXP, ClpCP, and ClpEP have clear roles in the virulence of both Gram-positive and Gram-negative bacteria, little is known about the role of HslUV (ClpYQ) in bacterial pathogenesis (16). In *Staphylococcus aureus*, a *clpYQ* deletion mutant is unable to form colonies at 45.4°C but grows normally at temperatures ≤45°C, suggesting that ClpYQ has a minor role in the heat shock response (29). In addition, the *S. aureus clpYQ* mutant is resistant to H_2_O_2_-induced oxidative stress and is as virulent as its parent in a murine skin abscess model (29). In contrast, *clpX* and *clpP* deletion mutants of *S. aureus* are highly attenuated in the skin abscess model, likely due to their control of several major staphylococcal virulence factors (30). In *Bacillus subtilis*, deletion of *clpYQ* has no effect on bacterial growth at temperatures as high as 50°C but severely impairs swarming motility (31). To our knowledge, our study is the first attempt to examine the role of HslUV in the pathogenesis of a gram-negative bacterium in humans.

Although *H. ducreyi* clearly encounters oxidative stress in pustules, we have never measured the temperature of infected sites. The skin surface temperature of the upper arm in healthy volunteers measured under controlled environmental conditions is 32.6 ± 0.9°C (32), a temperature range that should permit the growth of *H. ducreyi*. However, in the model, most of the inoculum is delivered to the deep dermis (12), which may have a higher temperature than that of the skin surface, especially when the dermis becomes inflamed. Thus, whether *H. ducreyi* faces heat stress in the dermis is unclear.

One limitation of this study is that once we determined that *hslUV* was dispensable for virulence in humans, we did not explore whether the gene products encoded by *hslUV* contribute to proteasome activity in *H. ducreyi*. A second limitation is that the duration of infection in the human challenge model is restricted to the pustular stage of disease. Since 2010, we have only tested unmarked deletion mutants in this model; we do not have IRB or FDA approval for competition experiments, which usually use antibiotic-resistant and sensitive strains. Although we cannot determine whether the *hslUV* mutant is less fit than the parent strain or whether HslUV contributes to the ulcerative stage of disease, our study reinforces the idea that pathogenic bacteria usually contain redundant systems to counteract stresses induced by the host immune response *in vivo*.

## MATERIALS AND METHODS

### Bacterial strains and culture conditions

*H. ducreyi* strain 35000HP was described previously (33); 35000HP∆*hslUV* was derived from dedicated human challenge stocks of 35000HP with minimal passage. Both strains were grown on chocolate agar plates supplemented with 1% IsoVitaleX at 33°C in 5% CO_2_. For the human challenge experiments, *H. ducreyi* was grown in GC broth supplemented with 1% IsoVitaleX, 5% heat-inactivated fetal bovine serum, and 50 µg/mL hemin at 33°C as previously described (33).

### Construction and characterization of 35000 HP ∆*hslUV*

35000 HP ∆*hslUV* was constructed by HiFi Assembly as previously described (21, 22). Briefly, three DNA fragments, a spectinomycin cassette flanked by flippase recognition target (FRT) sites from pRSM2832, ~500 bp of sequence upstream of *hslUV* that included the start codon and ~500 bp of sequence downstream of *hslUV* that includes the last seven codons, were PCR amplified by Phusion polymerase (Thermo Fisher) using primers designed by the NEBuilder program (New England Biolabs [NEB]; Table S2). Fragments were assembled and inserted into BamHI-digested pRSM2072 using HiFi Assembly Master Mix (NEB). The resulting plasmid—pRSM2072-∆*hslUV*—was transformed into *E. coli* NEB-10β, which lacks methylases. Purified plasmid was then electroporated into 35000HP. A spectinomycin-resistant colony was then selected and electroporated with pRSM2975, which encodes a tetracycline-inducible flippase. Following induction with anhydrotetracycline, clones that were spectinomycin sensitive were selected. The resulting mutant, 35000HP∆*hslUV,* was subjected to whole-genome sequencing. Sanger sequencing was used to confirm the deletion and resolve any low-quality single nucleotide polymorphism calls.

When needed during mutant construction, media were supplemented with kanamycin (20 μg/mL for *H. ducreyi*; 50 μg/mL for *E. coli*), ampicillin (10 μg/mL for *H. ducreyi*; 50 μg/mL for *E*. c*oli*), or spectinomycin (200 μg/mL for *H. ducreyi*; 50 μg/mL for *E. coli*). Primer sequences used for the construction of the mutant are shown in Table S2.

### RNA isolation and RT-PCR

To determine the operon organization of *hslUV* and to determine whether *purL* was transcribed in 35000HP∆*hslUV*, RNA was isolated from aerobic mid-log phase cultures of 35000HP as previously described (22). The QuantiTect SYBR green master mix (Qiagen) and custom primers (Table S2) were used for RT-PCR. One nanogram of RNA was used per reaction. A reaction without reverse transcriptase served as a negative control, and purified 35000HP gDNA served as a positive control for each primer set.

### Oxidative stress and heat shock assays

Oxidative stress assays were done as previously described (25). Briefly, 35000HP and 35000HP∆*hslUV* were grown to mid-log (OD_660_ ~0.2) or stationary phase (~16 h); and treated with 0.2 mM or 2.0 mM paraquat (Sigma-Aldrich) or 2.0 mM H_2_O_2_ (Fisher Scientific) at 33°C for 1 h and quantitatively cultured. Heat shock assays were done as described (27) with the modification that mid-log or stationary phase cultures grown at 33°C were incubated at 40°C for 2 h and then quantitatively cultured. For both the oxidative stress and heat shock assays, percent survival was calculated by determining the ratio of CFU recovered after treatment to CFU recovered prior to treatment. Significant differences in percent survival from four independent experiments were determined using a mixed-model analysis of variance (ANOVA) followed by Tukey’s honestly significant difference test; an adjusted two-sided *P* value of <0.05 was considered statistically significant (28).

### Human inoculation experiments

Mutant versus parent comparison trials are double-blind dose ranging with a minimum of two stages (10). Six healthy adult volunteers (three males and three females; two Asian persons, one Black person, and three White people; mean age ± SD 31.7 ± 14.9) between the ages of 22–61 years of age enrolled in the study.

Stocks of 35000HP and 35000HP∆*hslUV* were prepared according to FDA guidelines under BB-IND no. 13064. Methods for preparation of the bacteria, determination of the EDD, inoculation, surface cultures, clinical observations, biopsies, and antibiotic treatment were done exactly as previously described (34). Clinical endpoints included resolution of infected sites, development of a painful pustule at any site, or 14 days of observation (10). As the outcomes of infected sites within a subject are not independent, comparisons of papule and pustule formation rates were performed using a logistic regression model with generalized estimating equations (GEE) (35). The GEE sandwich estimate for the standard errors was used to calculate 95% confidence intervals (95% CI) for the rates.

Colonies recovered from the inocula, surface cultures, and biopsies were tested for the presence of *hslUV* and *dnaE* using probes generated by specific primers (Table S2) exactly as previously described (22).

## SUPPLEMENTAL MATERIAL

Table S1 iai.00577-24-s0001.pdf *H. ducreyi* homologs of protein quality control systems found in *Haemophilus influenzae* and *Escherichia coli*.

Table S2 iai.00577-24-s0002.pdf Oligonucleotides used in this study.

ASM does not own the copyrights to Supplemental Material that may be linked to, or accessed through, an article. The authors have granted ASM a non-exclusive, world-wide license to publish the Supplemental Material files. Please contact the corresponding author directly for reuse.
