# Supplementary material for: The protein degradation system encoded by hslUV (ClpYQ) is dispensable for the virulence of Haemophilus ducreyi in human volunteers
Source: Infect Immun. 2025 Apr 10;93(5):e00577-24. doi: 10.1128/iai.00577-24 (PMC12070733; doi:10.1128/iai.00577-24)
Supplement: Table S1 — H. ducreyi homologs of protein quality control systems found in Haemophilus influenzae and Escherichia coli. [file iai.00577-24-s0001.pdf]

Table S1. *H. ducreyi* homologs of protein quality control systems found in *Haemophilus influenzae* and *Escherichia coli*

Locus Tag	Gene Name	*H. influenzae*[a]	*E. coli*[a]
HD2006	*hslV*	65%/83%	65%/83%
HD2007	*hslU*	78%/88%	73%/87%
HD0221	*clpP*	76%/87%	73%/87%
HD0218	*clpX*	67%/82%	63%/77%
HD0565	*clpB*	77%/87%	73%/85%
HD0189	*dnaK*	88%/93%	82%/91%
HD1149	*lon*	72%/83%	68%/81%

[a] %identity / % similarity
