# Supplementary material for: The protein degradation system encoded by hslUV (ClpYQ) is dispensable for the virulence of Haemophilus ducreyi in human volunteers
Source: Infect Immun. 2025 Apr 10;93(5):e00577-24. doi: 10.1128/iai.00577-24 (PMC12070733; doi:10.1128/iai.00577-24)
Supplement: Table S2 — Oligonucleotides used in this study. [file iai.00577-24-s0002.pdf]

TABLE S2. Oligonucleotides used in this study

Primer (gene)	Purpose	Sequence
P1 (pRSM2072-*hslV* 5')	*hslUV* mutagenesis	GCGGCCGCTCTAGAACTAGTGAAACTCACGTGGAAATTGCCC
P2 (*hslV*-specR 3')	*hslUV* mutagenesis	ATCCCCGGAATCATGTTAAATCCTTCTTTTAGCAAAAACAG
P3 (*hslV*-specR 5')	*hslUV* mutagenesis	ATTTAACATGATTCCGGGGATCCGTCGACCT
P4 (specR-*hslU* 3')	*hslUV* mutagenesis	AGCGACTTAATGTAGGCTGGAGCTGCTTCGAAG
P5 (specR-*hslU* 5')	*hslUV* mutagenesis	TCCAGCCTACATTAAGTCGCTTTATTTTATAATTTGC
P6 (*hslU*-pRSM2072 3')	*hslUV* mutagenesis	TCGAATTCCTGCAGCCCGGGGATAAAACTCAAAATAATAAGCTAAACC
P7(hslV 5')	colony hybridization	GCGATGGACAAGCAACATTAG
P8 (hslU 3')	colony hybridization	TGAACACCTTCACGCGATAC
P9 (*dnaE* 5')	colony hybridization	AACGTTACCTTCAGCAAGCGGTTC
P10 (*dnaE* 3')	colony hybridization	GGCGTTTGGGATCGTCGAGTGTAT
P11 (*hslV*-*hslU* 5')	operon RT-PCR	GCTAAACAGATCGTTGCAGAAG
P12 (*hslV*-*hslU* 3')	operon RT-PCR	TAGCTTCAGTTTGGCCGATAA
P13 (*hslU*-*purL* 5')	operon RT-PCR	GGATGGTATTTCGTTTGATGCTAAT
P14 (*hslU*-*purL* 3')	operon RT-PCR	GAAATTCAGAGAGGGCTGGAG
P15 (*purL* 5')	*purL* RT-PCR	ACAGAAGTCTATCCAGCCAATC
P16 (*purL* 3')	*purL* RT-PCR	CTCTGGGTGTGGCATCATAATA
